# Silencing expression of PHF14 in glioblastoma promotes apoptosis, mitigates proliferation and invasiveness via Wnt signal pathway

**DOI:** 10.1186/s12935-019-1040-6

**Published:** 2019-11-27

**Authors:** Shuai Wu, Chen Luo, Fengjiao Li, N. U. Farrukh Hameed, Qiuyan Jin, Jie Zhang

**Affiliations:** 1 Glioma Surgery Division, Neurologic Surgery Department, Huashan Hospital, Fudan University, Shanghai, 200040 China; 2 Department of Human Anatomy and Histoembryology, Fudan University, Shanghai, 200433 China

**Keywords:** GBM, PHF14, Proliferation, Apoptosis, Invasiveness

## Abstract

**Background:**

The plant homeodomain (PHD) finger protein 14 (PHF14) is a vital member of PHD finger protein families. Abnormal expression of PHF14 has been identified in various cancers and is known to be implicated in the pathogenesis of tumors. This study investigates the role and the underlying mechanisms of PHF14 in GBM (glioblastoma multiforme).

**Methods:**

Tissue microarrays and public databases interrogation were used to explore the relationship between the expression of PHF14 and GBM. Three stable PHF14-silenced cell lines (U251, U87MG and A172) were constructed to assess the biological functions changes of GBM cells in vitro. In addition, tumorigenicity in vivo was also performed using U87MG cell line. To understand the mechanism of action of PHF14, RNA-Seq, qRT-PCR, Western blot, IC50 assay and subsequent pathway analysis were performed.

**Results:**

Our results showed that the expression of PHF14 was upregulated in glioma, especially in GBM. Overexpression of PHF14 translated to poor prognosis in glioma patients. In vitro assays revealed that silencing expression of PHF14 in glioma cells inhibited migration, invasiveness and proliferation and promoted cell apoptosis. Animal assay further confirmed that over-expression of PHF14 was a dismal prognostic factor. Analysis based on RNA-Seq suggested a PHF14-dependent regulation of Wnt signaling networks, which was further validated by qRT-PCR, Western blot and IC50 analysis. In addition, the mRNA expression of several key markers of EMT (epithelial–mesenchymal transition) and angiogenesis was found to change upon PHF14 silencing.

**Conclusions:**

Our data provide a new insight into the biological significance of PHF14 in glioma and its potential application in therapy and diagnosis.

## Background

Glioblastoma multiforme (GBM) is the most common and lethal primary tumor of the central nervous system (CNS) [1]. Despite aggressive therapy, the median overall survival is usually around 15 months [2, 3]. The mechanisms underlying the pathogenesis of GBM are complex, and epigenetic abnormalities play an important role [4–7].

The Plant Homeodomain (PHD) finger is a protein motif capable of epigenetic regulation via reading histones states [8]. PHD finger plays a key role in the recognition and translation of histone modification marks primarily by differentially recognizing methylated [9, 10] or unmodified [11, 12] lysine, directly contributing to gene transcription activation and silencing. Notably, the dysregulation of PHD finger family proteins (PHF) (e.g., PHF1, PHF6, PHF3, PHF10 and PHF11) can result in immunodeficiency [13, 14], cancer [15, 16] or neurological disorder [17, 18] via incorrect gene transcription.

The PHD finger family is closely associated with GBM. PHF6 has been reported as a potential TMZ-sensitizing biomarker in resistant GBM cells [18]. Similarly, PHF19 has been found to be upregulated in human glioma cells and can drive the proliferation of glioma cells [19]. PHF5A has been shown to be related to the expansion of GBM stem cells (GSC) [17]. However, the role of PHF14 remains to be explored in glioma.

PHF14, a member of the PHD finger protein family, is coded by a highly conserved gene found in the 19p13.2 chromosomal region [20]. PHF14 has been described as a modulator for mesenchymal cells proliferation in alveolar tissues [20, 21], as well as a novel hypoxia-sensitive epigenetic regulator in cell proliferation [22]. However, the biological function of PHF14 in tumors differs according to different cancer types. While PHF14 depletion has been shown to suppress cell proliferation in lung cancer cells and bladder cancer cells, its overexpression constrained BTC cells growth and improved prognosis in colon cancer [22–26]. The role of PHF14 in GBM has not been adequately described. It has been reported that mesenchymal gene expression signature in GBM is highly associated with increased invasiveness and poor prognosis [27]. Besides, hypoxia necrosis is one of the typical pathological characteristics of GBM [4]. Therefore, we suspected that PHF14 may be closely related to the malignant nature of GBM. In this research, we screened PHF14 as a biomarker related to GBM prognosis, and then performed in vitro assays to evaluate its roles in U251, U87MG and A172 cell apoptosis, proliferation and invasiveness. RNA-Seq analysis and certificating Western blot were performed to reveal the underlying mechanisms of the overall biological process.

## Methods

### Tissue specimen and tissue microarray construction

Immunohistochemical stainings for PHF14 expression were performed on 3 tissue microarrays comprising 105 glioma samples and 5 normal brain samples. For every tissue microarray, there are 35 glioma samples with same WHO grade and 5 normal brain samples, for each sample, two spots were repeated and placed on one tissue microarray. All tumor samples were collected from patients who were treated in Department of Neurosurgery, Huashan hospital, Fudan University. All tumors were classified and graded according to the WHO classification of tumor of the CNS [28]. Within 105 glioma samples, 35 was WHO grade II, 35 was WHO grade III and 35 was WHO grade IV. Five normal brain samples were collected from volunteer donors. For semi-quantitative analysis of PHF14 staining intensity, we graded PHF14 staining arbitrarily from 0–3 based on the fraction of moderately or strongly PHF14-positive cells (0, < 20%; 1, < 20–50%; 2, 50–80%; 3, > 80% PHF14-positive cells, respectively). Statistics were performed using one-way ANOVA.

### Immunohistochemistry

4 μm tumor tissue microarrays were baked at 60 °C for 30 min before conventional dewaxing and rehydration steps. Then the array slides were incubated in 0.3% of H_2_O_2_ for 5 min to quench endogenous peroxidase, boiled in 10 mM of sodium citrate at pH 6.0 for 30 min to retrieve antigen, blocked in PBS with 1% normal goat serum and 0.2% Triton X-100 for 1 h, stained with anti-PHF14 antibody (ThermoFisher Scientific, Cat# PA5-51406, 1:150) in blocking buffer overnight at 4 °C, and incubated with goat anti rabbit biotinylated IgG (Vector Laboratories, Cat# PK-4001) in blocking buffer for 1 h. Signal amplification and detection was performed according to manufacturers’ instructions using Vectastain ABC HRP kit (Vector Laboratories, Cat# PK-4001) and DAB Peroxidase Substrate kit (Vector Laboratories, Cat# SK-4100). The array slides were counterstained with Vector Hematoxylin (Vector Laboratories, Cat# H-3401) for 3 min, rinse with tap water, dehydrated and mounted using Permount (Fisher Scientific). Slides were digitally scanned by Pannoramic MIDI (3D HISTECH, Hungary) at 20× magnification.

### Database interrogations

Publicly available microarray, RNA sequence and clinical data of patients with glioma were acquired from the REpository for Molecular BRAin Neoplasia DaTa (REMBRANDT) using the data set available on 25 May 2018 (GSE108476) [29], the FRENCH using the data set available on 26 April 2010 (GSE16011) and from The Cancer Genome Atlas (TCGA, https://cancergenome.nih.gov) using the data set available on 15 December 2012 [30]. The mRNA expression data of PHF14 from REMBRANDT (n = 550) and FRENCH (n = 276) and were queried via the R2 microarray analysis and visualization platform (https://hgserver1.amc.nl/cgi-bin/r2/main.cgi). As for the mRNA expression data from TCGA (n = 1056), we downloaded from R2 and Xena (https://xenabrowser.net/). The molecular pathology data of these patients in TCGA were acquired from cBioPortal (https://www.cbioportal.org/). LGG refers to WHO Grade II and III gliomas. When we did Kaplan–Meier survival analysis using publicly available data in lower grade glioma (LGG) and GBM, the median mRNA expression level was regarded as cut-off value. As for the correlation analysis between PHF14 and PRC2 (Polycomb repressive complex 2), GEPIA (Gene Expression Profiling Interactive Analysis) was introduced (http://gepia.cancer-pku.cn/index.html) [31].

### Cell culture

Human glioma cell lines U251, U87MG, and A172 were purchased from Cell Bank of Type Culture Collection of Chinese Academy of Sciences (Shanghai, China). Cells were cultured in Dulbecco’s modified Eagle’s medium (Hyclone, Cat# SH30243.01B) containing 10% FBS (BI, Cat# 04-001-1A) at 37 °C in a humidified incubator containing 5% CO_2_.

### Lentiviral and stably transfected cells constructs

The following PHF14-targeted short hairpin RNAs were designed and synthesized by Genechem Co. Ltd (Shanghai, China). The recombinant lentivirus of small interfering RNA targeting PHF14 (PHF14-siRNA-lentivirus) and control lentivirus (GFP-lentivirus) were commercially prepared. Briefly, a lentivirus transfer vector (GV248) was constructed. The vector contained an ampicillin resistant gene and an enhanced green fluorescent protein gene. Expression of shRNA was driven by a U6 promoter. Packaging of viruses was performed by transient transfection of 293T cells with a transfer plasmid and 3 packaging vectors: pGC-LV, pHelper 1.0, and pHelper 2.0. Three days after transfection, lentiviral particles were collected, filtered, and concentrated by ultracentrifugation at 50,000*g* for 2.5 h at 4 °C. U251, A172 and U87 cell lines were infected with sh-PHF14. After 48 h, U87MG, U251 and A172 GBM cells were exposed to puromycin 0.5 μg/ml, 2 μg/ml and 2 μg/ml, respectively (Yeasen Biotech Co., Ltd, Cat# 60210ES25). The medium and puromycin would be changed everyday. One week later, cells were harbored and detected the expression level of PHF14. The targeting sequence of the short hairpin RNA: ggGATGTGCAGAGCCTATTTC.

### Clonogenicity formation assays

For clonogenic formation assay, 500 cells were seeded into per well of 6-well plates. The medium was changed every 3 days. After 10 days, cells were fixed by cold 4% paraformaldehyde and then stained using 1% crystal violet solution (BBI Life Science, Cat# F409FA0003). Colony consisted more than 50 cells was regarded as a single colony. The colony number was counted manually at 10× magnification.

### EdU assays

EdU Apollo 567 Cell Tracking Kit (Rib-bio, Guangzhou, China, Cat# C10310-1) was used to evaluate the proliferation of glioma cells. PHF14 scramble and silencing cells (1 × 10^4^/well) were seeded into 96-well plates and incubated at 37 °C overnight. Then 5-ethynyl-20-deoxyuridine (EdU, 200 μM) was added and incubated for 2 h at 37 °C. Cells were fixed with cold 4% paraformaldehyde for 20 min and then treated with 0.5% Triton X-100 for 10 min at room temperature. After that, washed with PBS for three times, and incubated with 100 μl of Apollo reagent for 30 min. Nuclei were then stained with Hochest 33342. The percentage of EdU-positive cells was calculated based on counts from 500 cells in three independent experiments.

### Migration and invasion assays

Migration and invasion of glioma cells was measured by transwell assay. For migration, 2.5 × 10^5^ cells in 1.0 ml serum-free Dulbecco’s modified Eagle’s medium were added to each transwell insert (24 wells, 8 mm pore size; BD Biosciences, USA, Cat# 3428). After incubation for 24 h, the cells in the upper membrane of insert were removed with a cotton swab and the cells adhering to the lower membrane of the inserts were fixed in ice-cold methanol at 4 °C and stained with 1% crystal violet. Quantification of cell migration was expressed as the mean count of stained cells in 5 random fields of each membrane under light microscope (10× objective lens). The invasion potential of glioma cells was evaluated by Transwell-Matrigel system. The culture upper inserts were coated with Matrigel (BD Matrigel, USA, Cat# 356234), the subsequence processes were carried out as Transwell assay. All the experiments were performed in triplicates.

### Three-dimensional (3D) tumor spheroid invasion assay

Glioma spheroids were generated by incubating 1000 cells in ultra-low attachment (ULA) 96-well round bottom plates (Corning, USA, Cat# 4515) with 10% Matrigel (BD Matrigel, USA, Cat# 356234) solution in 200 μl DMEM containing 10% FBS. After centrifuging the plate at 300×*g* for 3 min at 4 °C, transfer the plate to an incubator at 37 °C and allow the matrigel to solidify. 1 h later, 100 μl DMEM containing 10% FBS was added to each well. After 7 days’ incubation in 37 °C incubator, the plate was scanned under light microscope. The image was afterwards analyzed by ImageJ (https://imagej.nih.gov/ij/). The invasion area was outlined and measured using the substract background tool.

### Quantitative reverse transcription polymerase chain reaction

Total RNA was extracted from the cells using Trizol reagent (Takara, Japan) according to the manufacturer’s protocol. Total RNA (0.5 μg) was reverse-transcribed by PrimeScript™RT Master Mix, using Random 6 mers and Oligo dT Primer (Takara, Japan, Cat# RR036A), and the resulting cDNA was used as a template in qRT-PCR using a standard SYBR premix Ex Taq (Takara, Japan, Cat# RR420A) on the Real-Time PCR Detection System (ABI, USA). GAPDH served as the internal control, and experiments were conducted in triplicate. Cycling conditions: 95 °C for 30 s, 40 cycles of 95 °C for 5 s, 60 °C for 34 s, followed by a melting curve analysis to confirm specificity of the primers. Technical triplicates were performed and ∆Ct was used for subsequent analysis. The primers used are shown in Additional file 1: Tables S1.

### Immunoblot analysis

Protein lysates were denatured and separated on 10% polyacrylamide gels. After transfer to polyvinylidene fluoride (PVDF) membranes (Roche Applied Science), membranes were blocked in 5% non-fat milk power for 1 h at room temperature and incubated with primary antibodies overnight at 4 °C. After several times of wash with TBST, membranes were incubated with secondary antibody for 1 h at room temperature. Finally, the membranes were visualized using the Tanon 5200 Western Blotting Detection System (Tanon, China). Primary antibodies used were anti-PHF14 (ThermoFisher Scientific, Cat# PA5-72775, 1:1000) and anti-β-Catenin (CST, Cat# 9587T, 1:1000) and anti-Phospho-β-Catenin (CST, Cat# 4176T, 1:1000). As for second antibodies, anti-rabbit IgG (Abcam, Cat# 7074, 1:10,000) and anti-mouse IgG (Abcam, Cat# 7076, 1:10,000) were used.

### Immunofluorescence staining (IF)

Cells were cultured on coverslips in 6-well plate overnight at 37 °C incubator. Then cells were fixed with 4% cold paraformaldehyde and permeabilized with 0.5% Triton X-100, blocked with 3% bovine serum albumin (Servicebio, Cat# G5001) for 30 min at room temperature. After washing with PBS for 3 times, cells were then incubated with primary antibody against PHF14 (ThermoFisher Scientific, Cat# PA5-72775, 1:200) at 4 °C overnight. Cell nuclei were stained by DAPI (Servicebio, Cat# G1012). The cytoskeleton was visualized through staining with anti-stain 488 phalloidin (Servicebio, Cat# G1028-Green) according to manufacturer’s instructions. Cy3 conjugated goat anti-rabbit IgG antibody (Servicebio, Cat# GB21303, 1:300) was used to detect the primary antibody. Finally, the slides were scanned using Pannoramic MIDI (3D HISTECH, Hungary).

### Flow cytometry

Flow cytometry analyses were performed using a LSR II flow cytometer (BD Biosciences). For apoptosis analysis, we performed AnnexinV-PE and 7-AAD staining according to manufacturer’s protocol of AnnexinV-PE/7-AAD Apoptosis Detection Kit APC (Yeasen Biotech Co., Ltd, China, Cat# 40310ES60). For starvation test groups, cells were washed with PBS and then digested using EDTA-free trypsin (Gibco, Cat# 15050065). After washing with PBS for twice, cells were resuspended using 1× binding buffer containing AnnexinV-PE and 7-AAD and incubated in dark at room temperature for 30 min. Cells were then analyzed within 1 h after staining. FlowJo (TreeStar Inc.) software packages were used for data analysis.

### RNA-Seq library preparation and sequencing

Total RNA was extracted by TRIzol reagent (Sigma-Aldrich, Cat# T9424-100ML). Concentration and integrity of the total RNA were assessed by ND-1000 Nanodrop (Thermo Fisher) and Agilent 2200 TapeStation (Agilent Technologies), respectively. 1 µg of total RNA was used for RNA sequencing library preparation using NEBNext^®^ Ultra™ RNA Library Prep Kit for Illumina (NEB, Cat# E7490L). cDNA library quality was finally assessed by Agilent 2200 TapeStation (Agilent Technologies). Libraries prepared from a negative control and a siPHF14 samples were pooled together and loaded into HiSeq Rapid SBS Kit V2 (200 cycle) (Illumina, Cat# FC-402-4021) for sequencing on Hiseq 3000 (Illumina).

### Animal assay

U87MG cells were digested into single-cell and resuspended in PBS. 8-week-old nude male mice (bablc/nu, SPF level) (SLAC laboratory animal Center, Shanghai, China) (n = 12) were divided equally into 2 groups (n = 6 to each group): U87-NC and U87-sh-PHF14. Cells (3 × 10^5^) were implanted into mice brains using a stereotactic apparatus (KDS310, KD Scientific; Holliston, MA, USA). Animals were closely followed and euthanized by cervical dislocation when they exhibited symptoms, such as severe hunchback posture, apathy, decreased motion or activity, dragging legs, or drastic loss of body weight. Tumors were excised, formalin-fixed, paraffin-embedded, and sectioned for hematoxylin and eosin (HE) staining and IHC of Ki67 (Abcam, Cat# ab15580, 1:200).

### IC50 assay

1 × 10^5^ U251 cells were plated and incubated in 96-well plate with 200 μl medium for each well. 24 h later, β-catenin inhibitor IWR-1 (MCE, HY-12238) were added to the wells with different concentrations gradient (0, 1, 2, 4, 8, 16, 32, 64 and 128 μmol/l). Four duplicate wells were set for each concentration. Next, culture the cells with different concentrations gradient of β-catenin inhibitor for 48 h at 37 °C. Then, change the medium with new DMEM containing 10 μl CCK-8 regent (YEASON, Cat# 40203ES60) for each well. Incubate the plate in 37 °C incubator for 1.5 h before measuring the optical density (OD) at the wavelength of 450 nm (Bio-Rad, USA). Cell viability rate was then calculated. Half maximal inhibitory concentration (IC50) was calculated using Dose–response analysis by Graphpad Prism 7. The experiment was repeated for three times.

### Statistical analysis

Statistical analyses were performed using two-tailed Student’s t-test. P values of < 0.05 were considered statistically significant. Graphs were generated with Prism 7 (GraphPad Software).

## Results

### PHF14 is overexpressed in GBM and upregulation of PHF14 is associated with poor prognosis

**Fig. 1:**
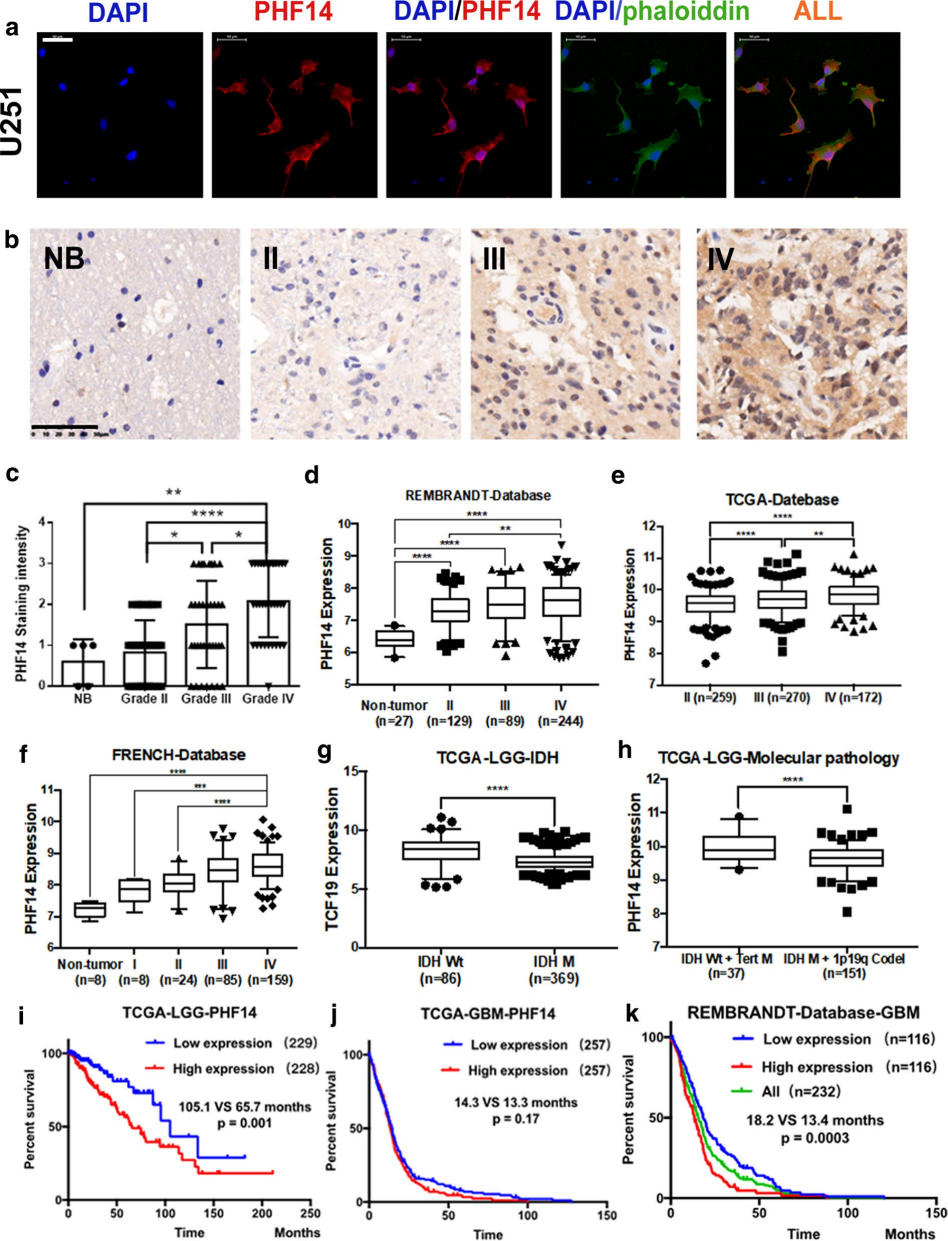
PHF14 is overexpressed in glioblastoma multiforme (GBM) and lower PHF14 expression correlates with better outcome in GBM and LGG. **a** The cellular location of PHF14 confirmed by immunofluorescence (Scale bar = 50 μm). **b** Representative stainings of PHF14 in normal brain, grade II, grade III and grade IV glioma (Scale bar = 50 μm). **c** Correlation of the staining intensity of PHF14 with glioma grade. **d**–**f** Correlation of PHF14 mRNA expression with increasing WHO glioma grade according to REMBRANDT, TCGA and FRENCH data. **g**, **h** The relationship between PHF14 mRNA expression and molecular pathology of glioma. **i**, **j** Upregulation of PHF14 is a dismal prognostic factor in LGG by TCGA database. **k** Upregulation of PHF14 is a dismal prognostic factor in GBM by REMBRANDT database (*P < 0.05; **P = 0.01; ***P < 0.001; ****P < 0.0001)

PHF14 is expressed both in nucleus and cytoplasm in U251 cells (Fig. 1a). Similar results were observed in U87MG and A172 cells (data not shown). By performing immunohistochemistry on three tissue microarrays, we found a positive correlation between glioma grade and the staining intensity of PHF14 (Fig. 1b, c).

To expand and validate our results in malignant gliomas, we performed public databases interrogation. The mRNA expression level of PHF14 significantly increases with increasing WHO glioma grade (Fig. 1d–f). The mRNA expression level of PHF14 was significantly higher in iso-citrate dehydrogenase (IDH) wild-type low-grade gliomas (LGG) (Fig. 1g). Compared to IDH-mutant LGG patients with 1p19q co-deletion, the expression of PHF14 was significantly higher in IDH wild-type patients with TERT-promoter mutation (Fig. 1h).

For correlation with clinical outcome, we subdivided samples into 2 groups by low (0–2) versus high (> 2) staining intensity of PHF14. Patients with higher staining intensity (> 2) of PHF14 had worse median overall survival in both GBM and LGG: (1) TCGA-LGG, PHF14 low (105.1 months) vs. high (65.7 months), P < 0.005; (2) TCGA-GBM, PHF14 low (14.3 months) vs. high (13.3 months), P = 0.17; (3) REMBRANDT-GBM, PHF14 low (18.2 months) vs. high (13.4 months), P < 0.001) (Fig. 1i–k).

### Knocking down the expression of PHF14 in glioma cell line decelerates proliferation and clonogenic growth

**Fig. 2:**
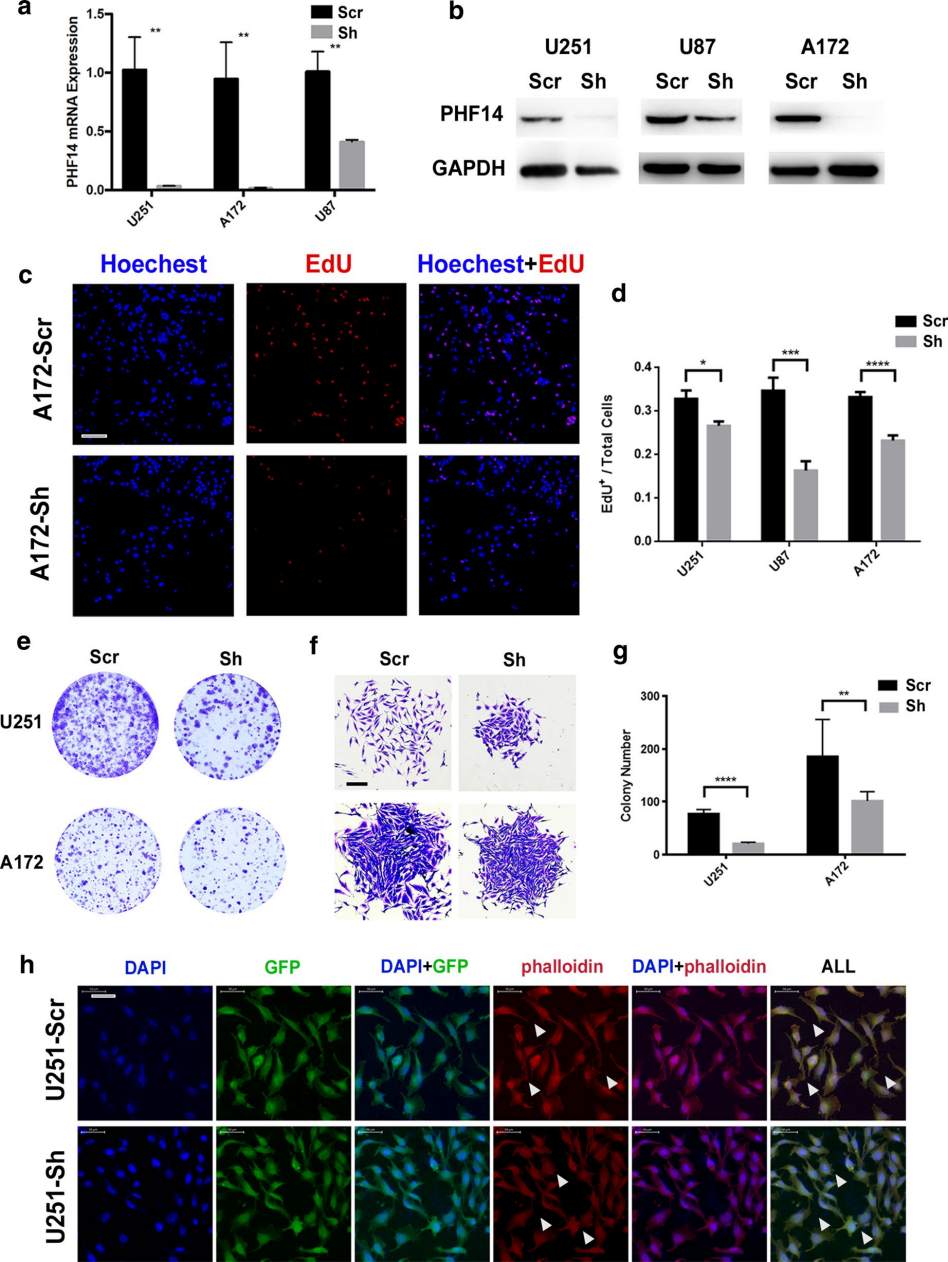
PHF14 silencing in glioma cell lines decelerates proliferation and clonogenic growth. **a**, **b** The knockdown efficacy of PHF14 examined in U251, U87MG and A172 cells by qRT-PCR (**a**) and Western blot (**b**). **c**, **d** Cellular DNA replication is mitigated after PHF14 knocked down. Ten thousand U87MG, U251 and A172 cells per well were seeded in 96-well plates overnight. EdU and Hoechest co-staining was performed on the second day to assess cellular DNA replication activities (**c**) (Scale bar = 100 μm). Values are counted as EdU + cells/total cells (**d**). **e**–**g** PHF14 silenced cells showed weaker proliferation and colony formation potential. 500 U251 and A172 cells per well were cultured in 6-well plates in Complete growth medium for 10 days and then stained by 1% crystal violet for colony formation assay (**e**) and single colony morphology (**f**) (Scale bar = 200 μm). The number of colonies containing over 50 cells were counted up for analysis (**g**). **h** Cell colonies were stained by Phalloidin-cy3 after 10 days of culture for assessment of actin filaments containment and cellular protrusions (Scale bar = 50 μm) (*P < 0.05; **P = 0.01; ***P < 0.001; ****P < 0.0001)

To study the biological function of PHF14 in glioma cells, we used lentiviral short hairpin RNA to construct the stable PHF14-silenced U251, U87MG and A172 glioma cell lines. Assessment of GFP co-expression revealed lentiviral transduction efficacies close to 100%. To confirm the knockdown efficiency of shRNA constructs, we performed qRT-PCR and western blot in U251, U87MG and A172 cells (Fig. 2a, b).

Subsequently, EdU assay showed that the growth of U251, U87MG and A172 cells decreased after PHF14 gene silencing (Fig. 2c, d, Additional file 2: Figure S1). Besides, after knocking down the expression of PHF14 gene, the number of colonies were reduced (Fig. 2e, g). Single colonies were also smaller and more compact, and cellular protrusions were reduced in size and number (Fig. 2f). By using Phalloidin (a specific marker of F-actin) staining, PHF14-silenced U251 (Fig. 2h) and A172 cells showed defects in producing filaments. Similar data were observed in U87MG (data not shown).

### PHF14 gene silencing enhances apoptosis and inhibits migration and invasion of glioma cells in vitro

**Fig. 3:**
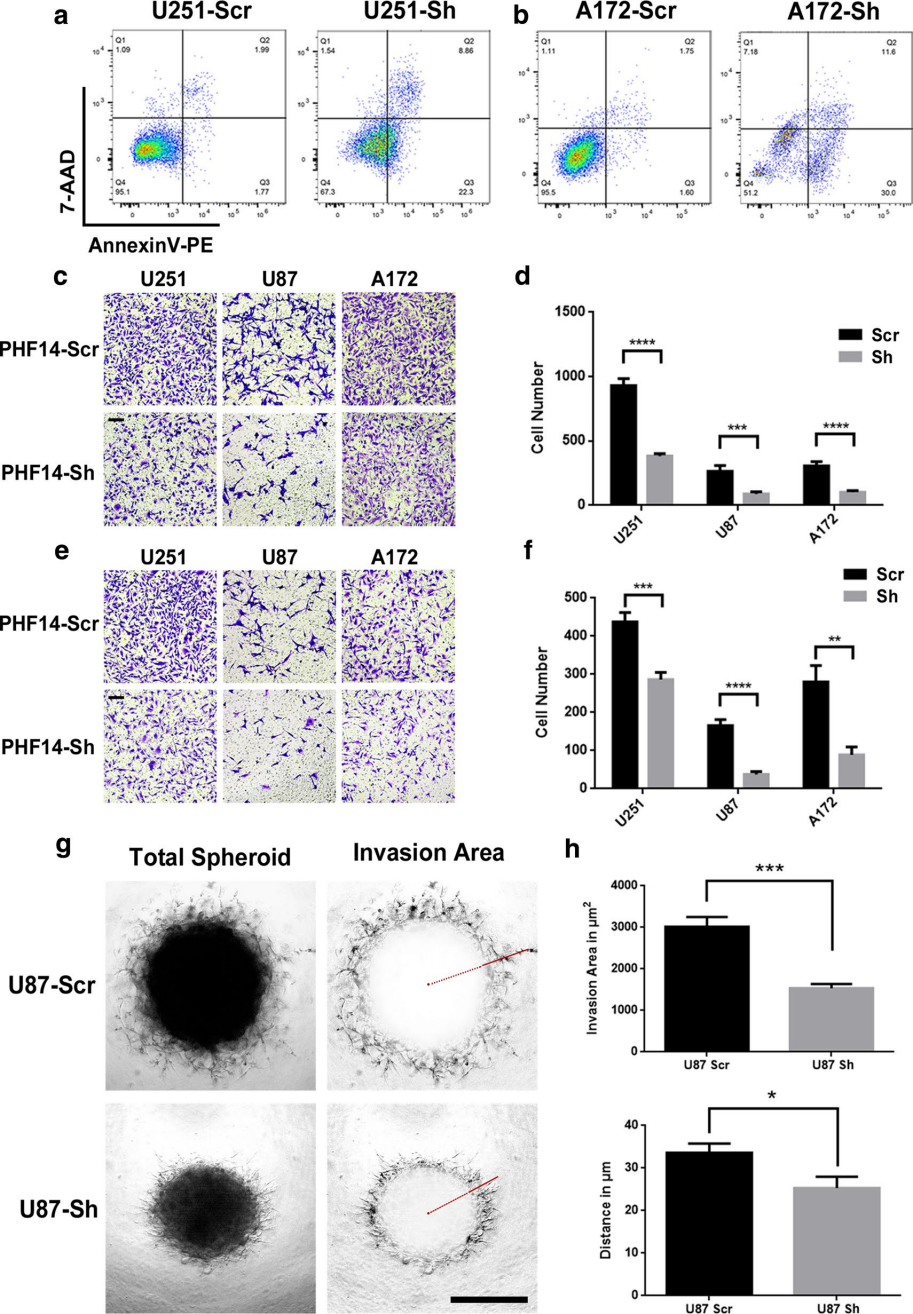
PHF14 gene silencing enhances apoptosis and inhibits migration and invasion of glioma cells in vitro. **a**, **b** Negative control and siPHF14 transfected U251 and A172 cells were stained with AnnexinV-PE and 7-AAD after cultured in serum-free medium for 7 days. Flow cytometric analysis was done afterwards for identification of live (both negative), early apoptosis (AnnexinV-PE-positive) and late apoptosis/dead cells (both positive). **c**–**f** Migration and invasion assay showed weaker migration and invasion potential of PHF14-silenced cells. 25 thousand U251, U87MG and A172 cells were seeded into the upper chamber of a transwell insert (**c**, **e**) (Scale bar = 100 μm). After 24 h of incubation, cells which had adhered to the lower membrane of the inserts were fixed, stained with 1% crystal violet and counted for analysis (**d**, **f**). For invasion assays, the upper inserts were pre-coated with Matrigel (**e**, **f**). **g**, **h** 1000 per well negative control and siPHF14 transfected U87 cells were placed in ultra-low attachment (ULA) 96-well round bottom plates with 10% Matrigel solution and cultured for 7 days. The invasion area outlined by ImageJ or the longest invasion distance (represented by red solid line) (**g**) were measured by quantitation afterwards (**h**) (Scale bar = 50 μm) (*P < 0.05; **P = 0.01; ***P < 0.001; ****P < 0.0001)

After 7 days of serum deprivation, the percentage of apoptotic (i.e. annexinV-positive) cells was 8.3-fold (31.16% versus 3.76%) higher in U251 short interfering PHF14 cells, and 12.4-fold (41.6% versus 3.35%) higher in A172 short interfering PHF14 transfected cells (Fig. 3a, b).

As mentioned above, the morphology of U251, U87 and A172 cells changed with knockdown of PHF14 in the clonogenicity formation assay. This change may influence the migration and invasion capabilities of glioma cells. The results of the transwell assay revealed that both the migration and invasion potential of U251-sh-PHF14, U87MG-sh-PHF14 and A172-sh-PHF14 cells were decreased relative to control cells both in transwell based experiment (Fig. 3c–f) and 3D spheroid model (Fig. 3g, h).

### PHF14 gene silencing attenuates tumorigenicity and delays the onset of deaths in vivo

**Fig. 4:**
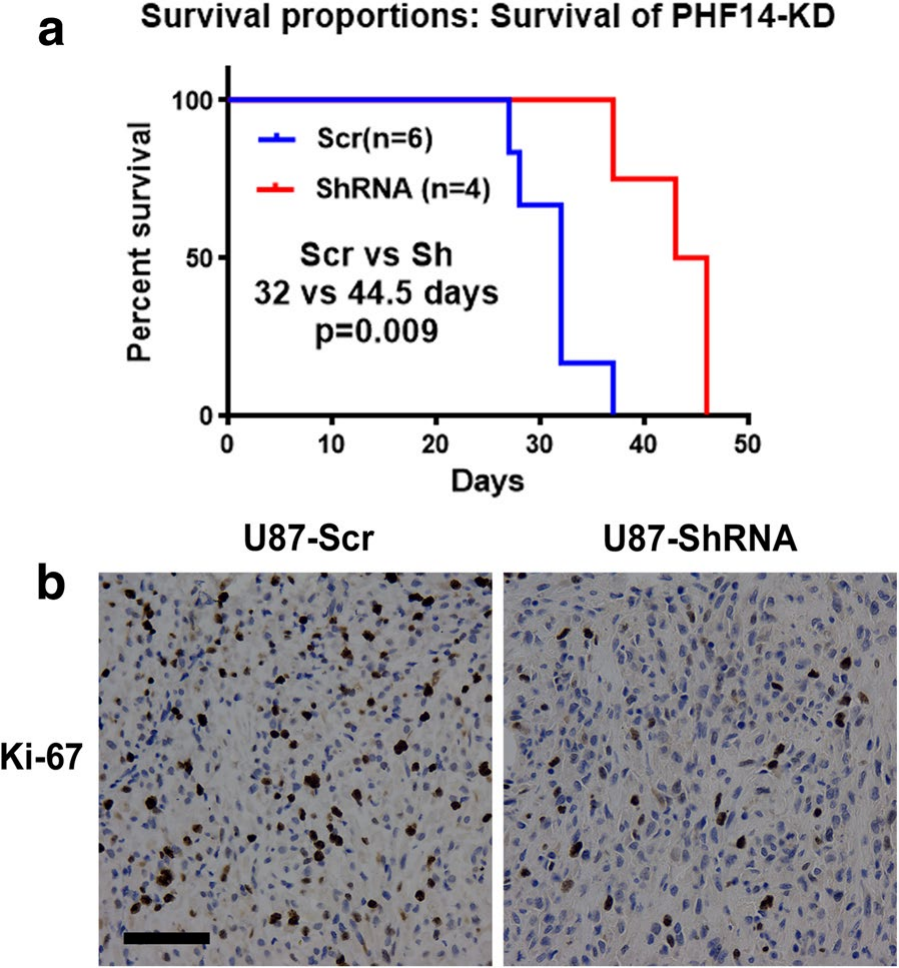
PHF14 silencing attenuates growth of glioblastomas and delays the onset of deaths in vivo. **a** 3 × 10^5^ U87MG scrambled or PHF14 silenced cells were implanted into 8-week-old nude mice (6 mice for each group). Once neurological disorders appeared, the animal would get sacrificed. The median overall survival of tumor bearing mice was prolonged from 32 days (n = 6) to 44.5 days (n = 4) after PHF14 was knocked down (P < 0.05). Two nude mice in PHF14 silencing group were excluded because no tumor was found after euthanized. **b** Ki-67 immunostainings of sliced brains of executed animals. PHF14 silenced cells represented weaker intensity, suggesting its lower proliferation rate (**b**, **c**) (Scale bar = 100 μm)

There is closely relationship between PHF14 and biological functions of GBM cell lines. To further revel the effect of PHF14 on GBM cells, animal assay was performed to investigate PHF14 expression on growth of glioblastomas in vivo. We implanted U87MG scrambled or PHF14 silenced cells into nude mice (6 mice for each group). As a result, two nude mice in PHF14 silencing group were excluded because no tumor was found after euthanized. The median overall survival of tumor bearing mice was prolonged from 32 days (n = 6) to 44.5 days (n = 4) after PHF14 was knocked down (P < 0.05) (Fig. 4a). Ki-67 staining was further performed on sliced brains of executed animals (Fig. 4b, c). The result showed that PHF14 silenced group represented weaker intensity, suggesting its lower proliferation rate in this group.

### An PHF14-dependent transcriptional regulation in GBM

**Fig. 5:**
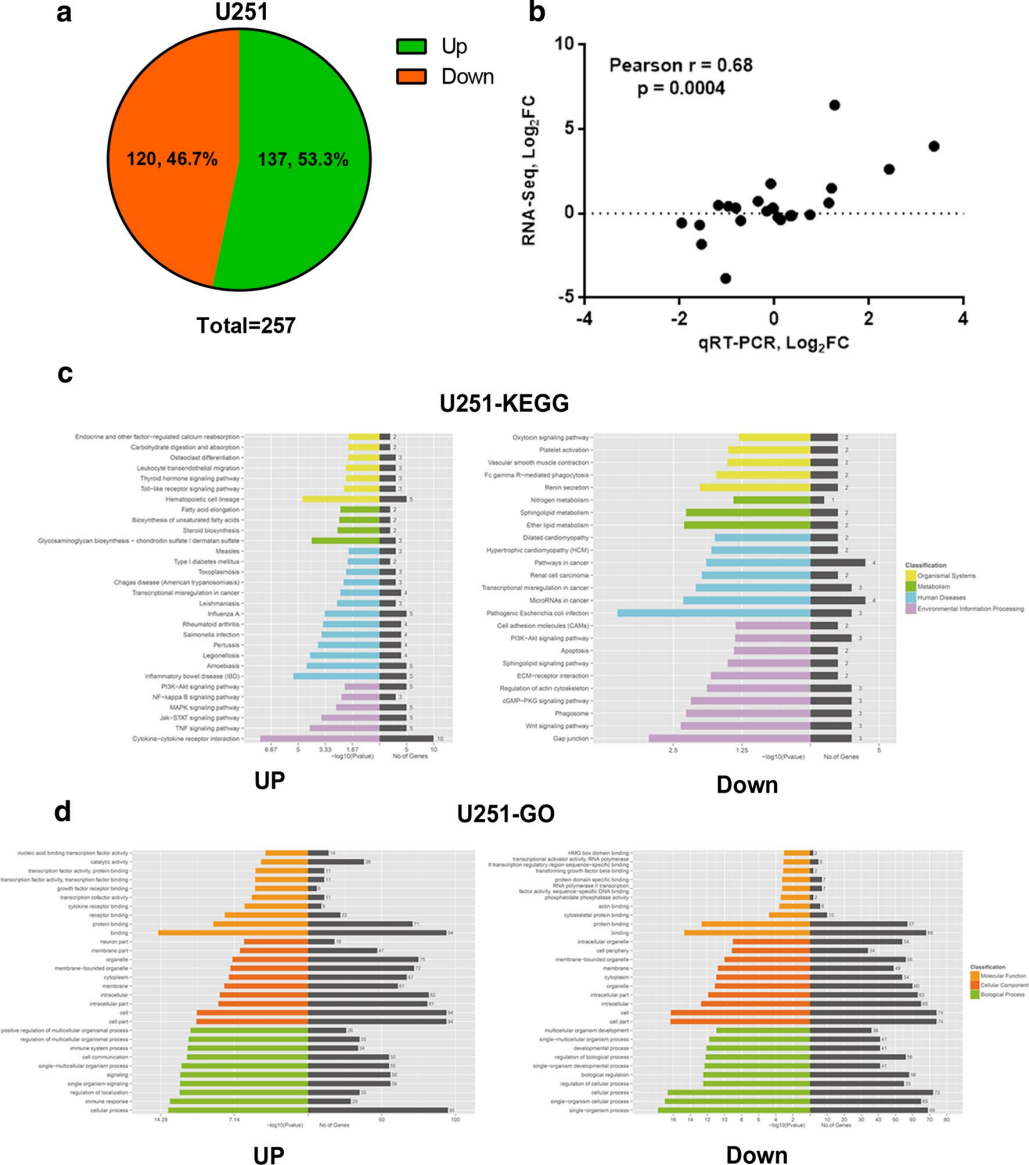
RNA sequencing analysis upon PHF14 depleted cells. **a** 137 upregulated and 120 downregulated genes (Log_2_ (Fold Change) ≤ − 1/≥ 1) were differentially expressed upon PHF14 knockdown with 5% False Discovery Rate (FDR). **b** Validation of the RNA sequencing analysis was performed by quantitative real time PCR. Pearson correlation of RNA Seq and qPCR is 0.68. **c**, **d** Gene ontology (GO) and KEGG pathway analyses results summarized that functional categories of genes significantly enriched upon PHF14 knockdown

To better understand the underlying mechanism of PHF14’s effect on glioma cells, we performed RNA sequencing analysis upon U251 negative control and shPHF14 transfected cells. We obtained a list of differentially expressed genes using edgeR package. Cut-off was set at a 1.0 − |Log_2_ (Fold Change)|. 137 upregulated and 120 downregulated genes were differentially expressed upon PHF14 knockdown with 5% False Discovery Rate (FDR) (Fig. 5a). Validation of the RNA sequencing analysis was performed by quantitative real time PCR. Pearson correlation of RNA Seq and qPCR is 0.68 (Fig. 5b). Figure 5c, d summarize the functional categories of genes significantly enriched upon PHF14 knockdown by using Gene Ontology (GO) and KEGG pathway analyses results. Among the enriched categories, a downward trend of Wnt (Wingless/Integrated) signaling pathways was observed on PHF14 silenced cells, which may suggest the further mechanism of PHF14 in GBM formation. The same trend was also found in U87MG cells (Additional file 3: Figure S2).

**Fig. 6:**
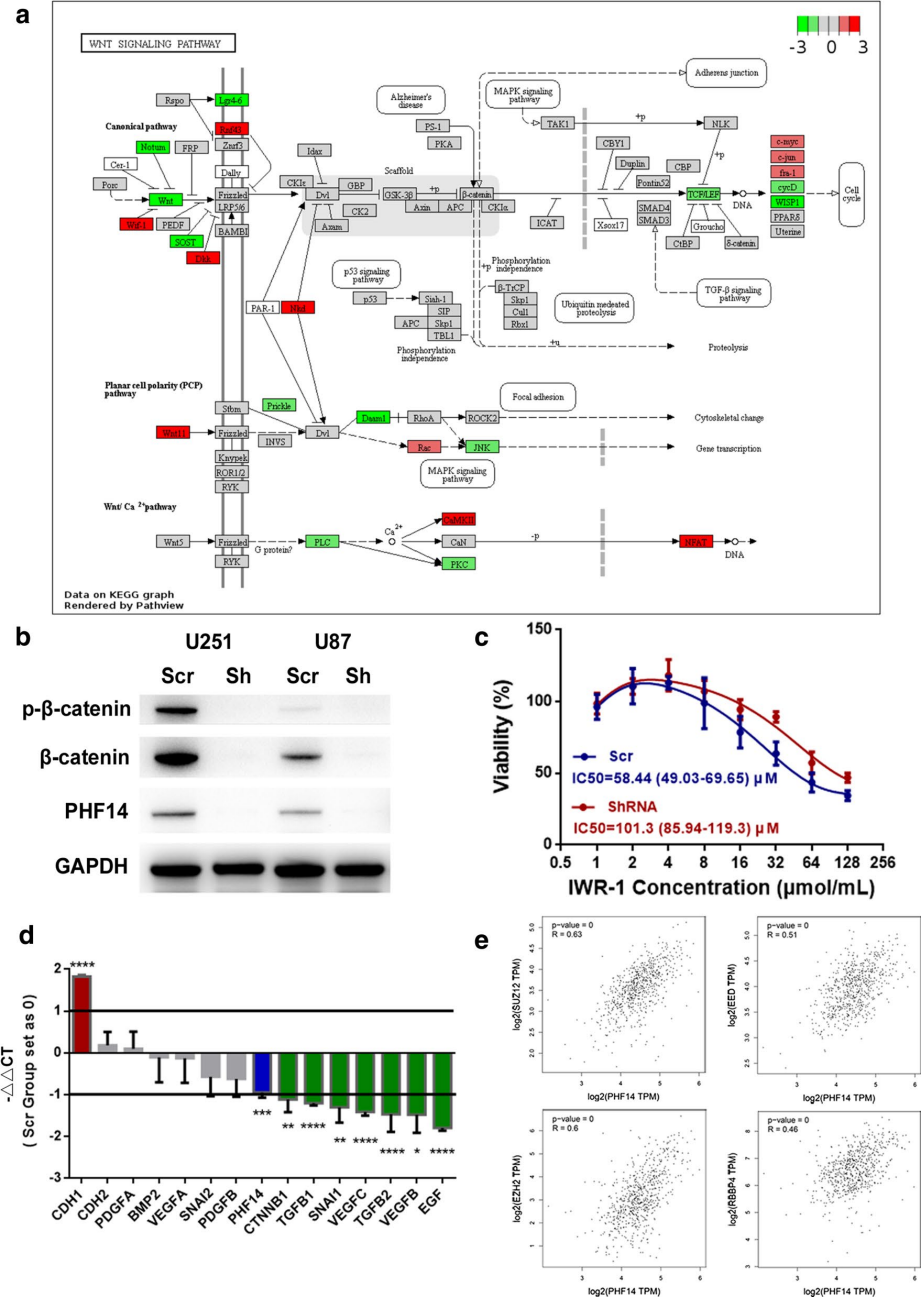
Differentially regulated transcriptional network in PHF14 depleted cells. **a** Pathway analysis proposed a PHF14-dependent regulation of Wnt-β-Catenin signaling pathway. Genes clusters were presented with red (− Log_2_FC ≥ 1) or green color (− Log_2_FC ≤ − 1). The color depth was correlated with the difference in gene expression. **b** Western blots showed that the levels of p-β-catenin and β-catenin decreased upon PHF14 knockdown. **c** PHF14-depleted cells showed higher tolerance to IWR-1-mediated inhibition of cell growth. U251 cells of both groups were treated with increased doses of IWR-1 for 48 h before determined by CCK-8 assay (450 nm). Results from three replicated experiment are shown, error bars represent standard deviation. IC50 value was determined by dose–response variable slope analysis using Graphpad Prism 7. **d** Quantitative RT-PCR indicated reduced mRNA level of several markers of EMT (epithelial–mesenchymal transition), PRO (Proliferation) and angiogenesis. **e** The high correlation of RNA expression between PHF14 and genes code for members of PRC2 including EED, EZH2, SUZ12 and RBBP4 was proved by correlation analysis. (*P < 0.05; **P = 0.01; ***P < 0.001; ****P < 0.0001)

To verify the role of PHF14 in the Wnt pathway, we first applied data obtained from RNA-seq to pathway analysis (https://pathview.uncc.edu/) (Fig. 6a). The result suggested a down-regulation of Wnt cascade after PHF14 silenced. We further confirmed altered signaling pathways using western blot analysis (Fig. 6b). β-catenin, which is a highly specific down-stream factor of Wnt signaling pathway decreased after knockdown of PHF14 both in U251 and U87MG cells. The conclusion was consistent with our RNA-Seq data.

Furthermore, as illustrated in Fig. 6c, CCK-8 was applied to detect cell viability with β-catenin selectively inhibited. It came out that cell growth in both group were decreased with the increased concentration of IWR-1 (the selective β-catenin inhibitor). However, PHF14-silenced group preserved a relatively higher survival rate than control group [IC50 value, ShRNA v.s. Scr: 101.3 (85.94–119.3) μmol/ml v.s. 58.44 (49.03–69.65) μmol/ml]. To claim how PHF14 affects invasion and migration capability of GBM cells, we performed qRT-PCR array analysis on markers of EMT and angiogenesis. We found that several EMT and angiogenesis related key factors were highly related to the expression of PHF14 (Fig. 6d). Using GEPIA correlationship analysis, we also found the expression of PRC2 complex members were highly correlated with PHF14, which may suggest the role of PHF14 in epigenetic regulation in this transcription network (Fig. 6e).

## Discussion

As a classic epigenetic regulator, the PHD finger is present in a variety of eukaryotic proteins that are involved in chromatin-mediated transcriptional regulation and chromatin dynamics [9, 10, 32]. They play a vital role in regulating cellular epigenetic events by acting as readers of histone modification marks, but not as a more classic identity of “writers” or “erasers” [6–12]. PHD proteins can also function as oncogenic proteins [33, 34]. Among them, PHF6 has been found to be upregulated in glioma, especially in TMZ resistant GBM, as a potential TMZ-sensitizing factor in resistant GBM cells [18]. Lu et al. found that PHF19 is also upregulated in human glioma cell lines and tissues, which is necessary for EZH2 activation and drives proliferation in these cells [19]. Hubert et al. suggested that plant homeodomain PHF5A is differentially required for GBM stem cell expansion [17]. In our study, PHD proteins were found to be highly related to the pathogenesis of glioma.

PHF14 is a chromatin binding protein containing four putative PHD fingers and two coiled-coil regions. It interacts with histones via its PHD1 and PHD3 domains, which indicate its potential role in epigenetic regulation [20]. The roles of PHF14 in different types of cancers are contradictory. PHF14 is overexpressed in lung and biliary tract cancer and downregulated in colon cancer [24–26]. We found PHF14 expression to be significantly higher in GBM than LGGs or normal brain. Supportively, the results from TCGA, REMBRANDT, and FRENCH databases verify PHF14 expression to be correlated with higher grades of malignancy and decreased overall survival in both GBM and LGG. Moreover, PHF14 expression level is significantly higher in LGG without IDH mutation than in LGG with IDH mutation. It is well known that the prognosis of glioma patients with IDH mutation and 1p19q co-deletion is relatively good when compared with those with IDH wide type plus Tert promoter mutation [35, 36]. LGG patients with IDH wild type plus Tert promoter mutation possess even higher PHF14 expression level than patients with IDH mutation and 1p19q co-deletion. PHF14 expression is also related to survival in GBM. Franceschi et al. reported that the mRNA expression pattern in GBM patients may define a particular molecular hallmark related to prognosis heterogenecity [37]. Our research found that higher expression of PHF14 in GBM is associated with poorer prognosis. The upregulation of PHF14 is really closely associated to the malignant nature of glioma, especially in GBM. Thus, we then focused our research on investigating the role of PHF14 in vitro. According to our results, upregulation of PHF14 could help GBM cells escape from apoptosis, enhance proliferation, migration and invasion ability. To further reveal the role of PHF14 in malignance of GBM, PHF14 overexpression rescue assays are also on the way to further confirm the role of PHF14 in GBM cells. We supposed overexpression of PHF14 may reduce apoptosis and increase proliferation and invasiveness in GBM cells.

The mechanism of PHF14 in tumorigenesis have been well-studied. PHF14 increases mitotic defects and DNA errors in lung cancer model [26]. It also induces G1-S phase arrest in squamous carcinoma [22]. In addition, PHF14 promotes tumor cellular epithelial–mesenchymal transition (EMT) and enhances the proliferation and invasiveness of bladder cancer [23]. Moreover, PHF14 could inhibit growth of mesenchymal cells in biliary tract cancer [24].

As an epigenetic regulator, the paradoxical function of PHF14 may be due to environmental stress and its functional condition. Considering the upregulation of PHF14 in glioma, especially in GBM, we presumed it to be a transcription regulator with repressing and/or activating roles at different genes during the tumorigenesis of GBM. To further reveal the biological function of PHF14 in GBM, we silenced the expression of PHF14 in three kinds of GBM cell lines. Our results showed that silencing of PHF14 in GBM cells promoted apoptosis upon nutrient deprivation, decelerated clonogenic growth, and inhibited migration and invasion both in transwell-based experimental and 3D spheroid model. The GO function and KEGG pathway analysis showed a tight relationship between PHF14 and Wnt signaling pathway. Wnt signaling pathway play a crucial role in CNS development by affecting self-renewal, differentiation and proliferation of neural stem/progenitor cells [38]. Consequently, abnormal activation of Wnt signaling can result in tumorigenesis and the differentiation of glioma stem cells (GSCs) into GBM cells [39, 40]. Our results showed the Wnt signaling pathway to be inhibited by silencing expression of PHF14, suggesting PHF14 involvement in the pathogenesis of glioma via Wnt/β-catenin signaling pathway. Studies which have utilized the selective β-catenin inhibitor IWR-1 have revealed its strong tumor suppressing activity [41]. While our study also observed the significant effects of IWR-1 on cell viability in U251 glioma cells, PHF14 depleted cells showed greater resistance to IWR-1 sensitization. It suggested an compensation mechanisms for the transfected cell line to maintain its growth rate which was independent of Wnt/β-catenin cascade. During the research, with the concentration of less than 8 μm, IWR-1 exhibited mild cell growth promotion in U251 cells. It needs to be further investigated in order to analyze potential aberrant β-catenin-regulation in malignant gliomas.

As we had observed enhanced apoptosis and decreased proliferation and invasion in PHF14 depleted cells, we also investigated into key markers of EMT and angiogenesis. As reorganization of the Cadherin-household is a hallmark of EMT as a driver of cell-to-cell contact loss, its expression is considered as negatively correlated with the malignancy potential of tumors [42, 43]. According to our results, PHF14-silenced GBMs showed relatively intrinsic CDH1 (E-Cadherin) expression and the downregulation of its transcriptional repressor SNAI1. Besides, some other factors were found to co-express with PHF14 in U251 cells, including angiogenic-associated markers (VEGF and EGF genes) and proliferation-associated markers (TGFB genes). We reasoned that the central role of Wnt signaling pathway in EMT process was consistent with alteration of these factors according to our results [44].

PHD fingers function by reading the N terminal tail of histone H3, especially H3K4 [45, 46] and H3R2 [47]. H3 mutations, some of which are dominant, have been deemed ‘oncohistones’ [48]. H3K27M was identified in 78% of diffuse midline gliomas and has been categorized as a separate pathological entity in the 2016 WHO classification [28]. Besides, H3Gpa34 V/R also occurs in paediatric glioblastomas [49]. To explore the mechanism of PHF14 in this process, we performed correlation analysis focusing on PRC2 complex. PHD-PRC2 sub-complexes which includes PHF1 and PHF19 have been proved to be a essential regulator of epigenetic activity [50]. Polycomb-like (PCL) proteins, which include PHF1, PHF3 and PHF19, are critical PRC2-associated factors and components of its sub-complexes [50–52]. These complexes are traditional epigenetic regulators, especially of H3 methylation, which mediates their crucial role on biological processes such as maintainance of proper proliferation and cell differentiation [50–52]. We found a significant positive relationship between PHF14 and PRC2. Interestingly, Wnt/β-catenin signaling pathway has also been shown to facilitate histone H3 trimethylation via physical association between Pygo2 (a highly specific downstream component of Wnt signaling pathway, also contains a highly conserved C-terminal PHD) and H3K4 [53]. Thus, our results also suggest an indirect function of PHF14 on the transcription machinery of histone H3. Further research is needed to describe the underlying mechanisms. Besides, PHF14 as a vital transcription factor, the target gene of PHF14 should be further explored.

## Conclusions

In summary, PHF14 expression correlates with glioma grades and patients’ survival. It regulates key malignant features in glioma models, including cell proliferation, colony formation, migration and invasiveness via Wnt signaling pathway. There is also an important relationship between PHF14 and PRC2, which indicates its potential roles in epigenetics. PHF14 could be a novel key molecule in the pathogenesis of gliomas and should be further explored as a putative therapeutic target.

## Supplementary information

**Additional file 1.** The primers used for our RT-qPCR assay.

**Additional file 2.** EdU assay showed that the growth of U251 and U87MG cells decreased after PHF14 gene silencing.

**Additional file 3.** GO and KEGG pathway analyses upon PHF14 knockdown in U87MG cells.

## Data Availability

All data and materials have been presented in the manuscript.

## Abbreviations

PHD: plant homeodomain
PHF14: plant homeodomain finger protein 14
GBM: glioblastoma
CNS: central nervous system
TMZ: temozolomide
FDR: false discovery rate
GO: gene ontology
PRC2: polycomb repressive complex 2
EMT: epithelial–mesenchymal transition
PRO: proliferation
