# Supplementary material for: Silencing expression of PHF14 in glioblastoma promotes apoptosis, mitigates proliferation and invasiveness via Wnt signal pathway
Source: Cancer Cell Int. 2019 Nov 27;19:314. doi: 10.1186/s12935-019-1040-6 (PMC6882144; doi:10.1186/s12935-019-1040-6)
Supplement: Supplementary file 1 — Additional file 1. The primers used for our RT-qPCR assay. [file 12935_2019_1040_MOESM1_ESM.docx]

| Gene Name | Direction | Sequence |
| --- | --- | --- |
| PHF14 | F | 5’-CTGTTCCCACTACGACAACCG-3’ |
|  | R | 5’-ACTACAGTAGGTCGCCAATCAT-3’ |
| GAPDH | F | 5’-AAAGACCCCTGCTTCCAGATT-3’ |
|  | R | 5’-TTCCCCACTCGTAAACACCAA-3’ |
| EED | F | 5’-CACCTACAAACACGCCAAATG-3’ |
|  | R | 5’-AACTCTGTTGCTTCCTACAGTTG-3’ |
| EZH2 | F | 5’-AATCAGAGTACATGCGACTGAGA-3’ |
|  | R | 5’-GCTGTATCCTTCGCTGTTTCC-3’ |
| SUZ12 | F | 5’-AGGCTGACCACGAGCTTTTC-3’ |
|  | R | 5’-GGTGCTATGAGATTCCGAGTTC-3’ |
| RBBP4 | F | 5’- ATGACCCATGCTCTGGAGTG-3’ |
|  | R | 5’- GGACAAGTCGATGAATGCTGAAA-3’ |
| RBBP7 | F | 5’- GGTGGCTTTGGTTCTGTAACA-3’ |
|  | R | 5’-ACGAGCACGGTTTACTTCTCC-3’ |
| MAPK8IP1 | F | 5’-ATCGCTTCGCCTCCCAATTT -3’ |
|  | R | 5’-ATCTCCGAGAGGTCTTCATCC -3’ |
| GFAP | F | 5’-CTGCGGCTCGATCAACTCA-3’ |
|  | R | 5’-TCCAGCGACTCAATCTTCCTC-3’ |
| PTEN | F | 5’-TGGATTCGACTTAGACTTGACCT-3’ |
|  | R | 5’-GGTGGGTTATGGTCTTCAAAAGG-3’ |
| ABCB5 | F | 5’-ATTGGAGTGGTTAGTCAAGAGCC-3’ |
|  | R | 5’-AGTCACATCATCTCGTCCATACT-3’ |
| CTNNB1 | F | 5’-AAAGCGGCTGTTAGTCACTGG-3’ |
|  | R | 5’-CGAGTCATTGCATACTGTCCAT-3’ |
| AEBP1 | F | 5’-ACCCACACTGGACTACAATGA-3’ |
|  | R | 5’-GTTGGGGATCACGTAACCATC-3’ |
| CAGE1 | F | 5’-AGCCCGCCTCTAATCCACT-3’ |
|  | R | 5’-GGAGGTTGGCTAGGGTTGAG-3’ |
| CXCR5 | F | 5’-CACGTTGCACCTTCTCCCAA-3’ |
|  | R | 5’-GGAATCCCGCCACATGGTAG-3’ |
| KHDC1L | F | 5’-GGACCCTGCCCGAAAACTTT-3’ |
|  | R | 5’-ACGTGTCATCAAGTCCGAAGA-3’ |
| AKT1 | F | 5’- CCTCCACGACATCGCACTG-3’ |
|  | R | 5’- TCACAAAGAGCCCTCCATTATCA-3’ |
| ABCB5 | F | 5’- ATTGGAGTGGTTAGTCAAGAGCC-3’ |
|  | R | 5’- AGTCACATCATCTCGTCCATACT-3’ |
| FUT2 | F | 5’- TCCCCTGGCAGAACTACCA-3’ |
|  | R | 5’- GGTGAAGCGGACGTACTCC-3’ |
| VEGFA | F | 5'-AGGGCAGAATCATCACGAAGT-3' |
|  | R | 5'-AGGGTCTCGATTGGATGGCA-3' |
| VEGFC | F | 5'-GAGGAGCAGTTACGGTCTGTG-3' |
|  | R | 5'-TCCTTTCCTTAGCTGACACTTGT-3' |
| VEGFB | F | 5'-GAGATGTCCCTGGAAGAACACA-3' |
|  | R | 5'-GAGTGGGATGGGTGATGTCAG-3' |
| EGF | F | 5'-TGGATGTGCTTGATAAGCGG-3' |
|  | R | 5'-ACCATGTCCTTTCCAGTGTGT-3' |
| PDGFA | F | 5'-GCAAGACCAGGACGGTCATTT-3' |
|  | R | 5'-GGCACTTGACACTGCTCGT-3' |
| PDGFB | F | 5'-CTCGATCCGCTCCTTTGATGA-3' |
|  | R | 5'-CGTTGGTGCGGTCTATGAG-3' |
| TGFB1 | F | 5'-GGCCAGATCCTGTCCAAGC-3' |
|  | R | 5'-GTGGGTTTCCACCATTAGCAC-3' |
| TGFB2 | F | 5'-CAGCACACTCGATATGGACCA-3' |
|  | R | 5'-CCTCGGGCTCAGGATAGTCT-3' |
| TGFB3 | F | 5'-ACTTGCACCACCTTGGACTTC-3' |
|  | R | 5'-GGTCATCACCGTTGGCTCA-3' |
| SNAI1 | F | 5'-TCGGAAGCCTAACTACAGCGA-3' |
|  | R | 5'-AGATGAGCATTGGCAGCGAG-3' |
| CDH1 | F | 5'-ATTTTTCCCTCGACACCCGAT-3' |
|  | R | 5'-TCCCAGGCGTAGACCAAGA-3' |
| SNAI2 | F | 5'-CGAACTGGACACACATACAGTG-3' |
|  | R | 5'-CTGAGGATCTCTGGTTGTGGT-3' |
| CDH2 | F | 5'-TCAGGCGTCTGTAGAGGCTT-3' |
|  | R | 5'-ATGCACATCCTTCGATAAGACTG-3' |
