# Supplementary figures and images for: Silencing expression of PHF14 in glioblastoma promotes apoptosis, mitigates proliferation and invasiveness via Wnt signal pathway
Source: Cancer Cell Int. 2019 Nov 27;19:314. doi: 10.1186/s12935-019-1040-6 (PMC6882144; doi:10.1186/s12935-019-1040-6)

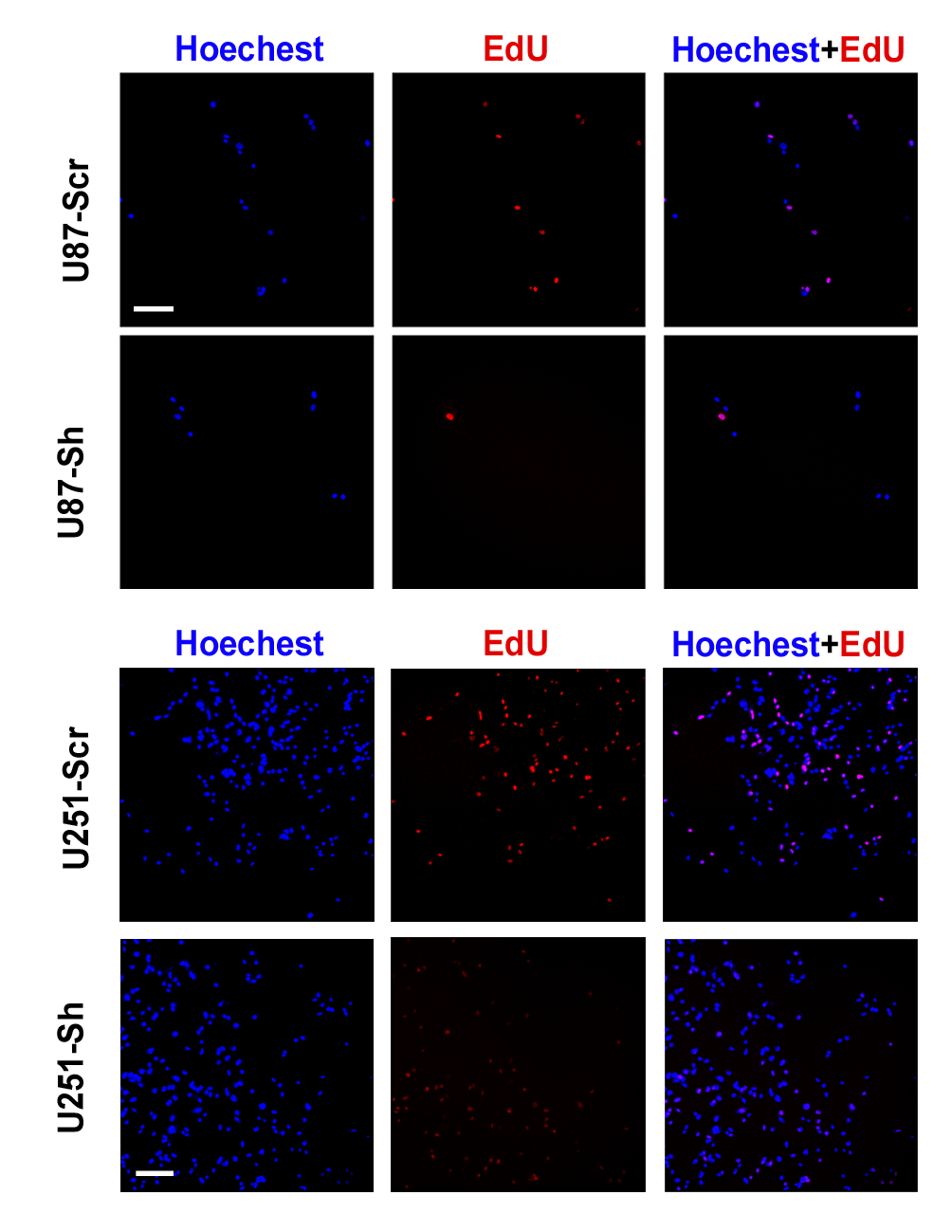

Supplement: Supplementary file 2 — Additional file 2. EdU assay showed that the growth of U251 and U87MG cells decreased after PHF14 gene silencing. [file 12935_2019_1040_MOESM2_ESM.tif]

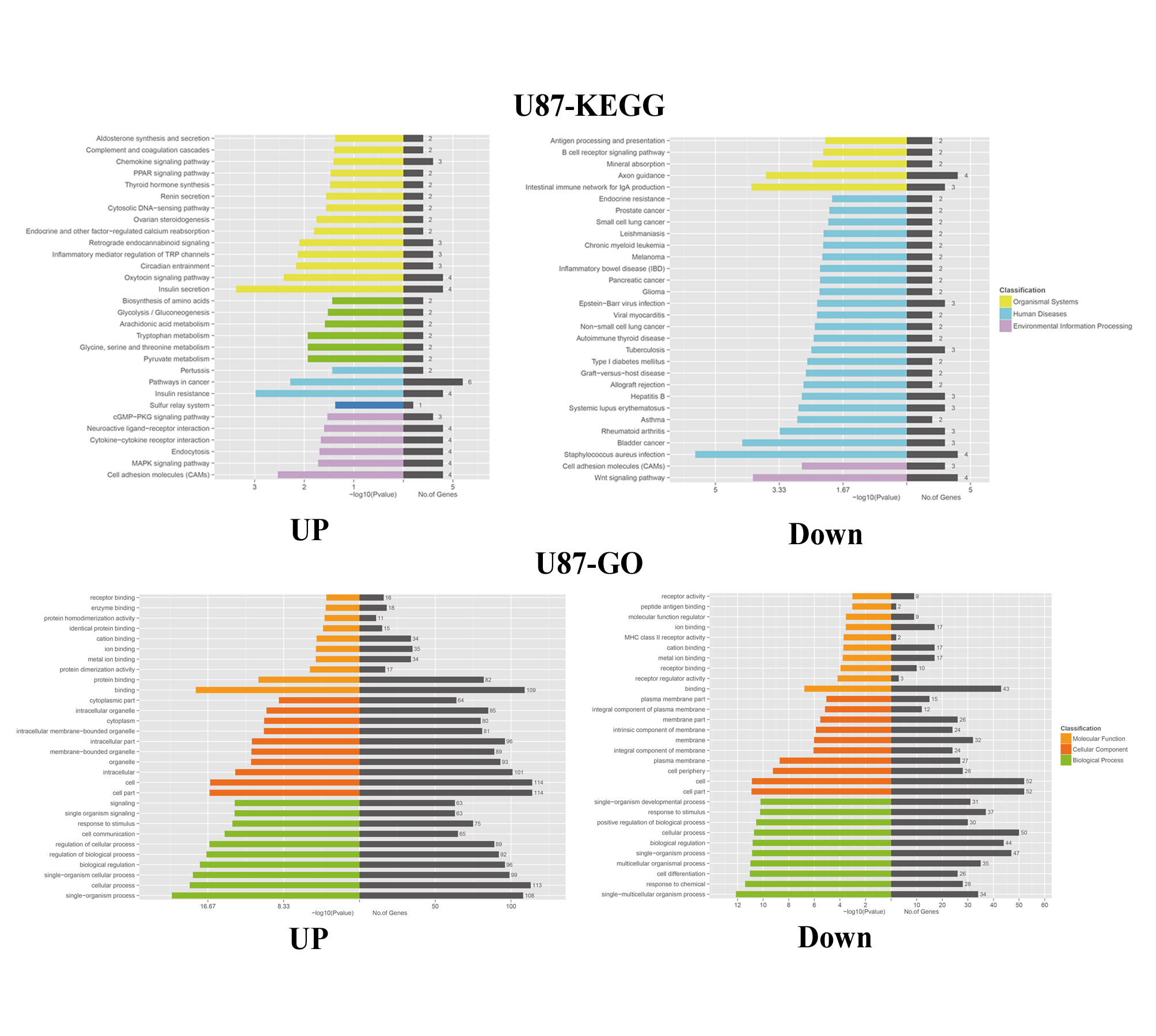

Supplement: Supplementary file 3 — Additional file 3. GO and KEGG pathway analyses upon PHF14 knockdown in U87MG cells. [file 12935_2019_1040_MOESM3_ESM.tif]
